# Structural Characterization and In Vitro Hypoglycemic Activity of a Polysaccharides Obtained from *Fructus arctii*

**DOI:** 10.3390/molecules30224403

**Published:** 2025-11-14

**Authors:** Pin Gong, Jiawei Gao, Hui Long, Haotian Gao, Wenjuan Yang, Jing Wang, Nan Li, Yanni Zhao, Huan Liu, Fuxin Chen

**Affiliations:** 1 School of Food Science and Engineering, Shaanxi University of Science and Technology, Xi’an 710021, China; 2 School of Biological and Pharmaceutical Sciences, Shaanxi University of Science and Technology, Xi’an 710021, China; 3 Xi’an Key Laboratory of Precision Nutrition and Functional Product Creation, Shaanxi University of Science and Technology, Xi’an 710021, China; 4 School of Chemistry and Chemical Engineering, Xi’an University of Science and Technology, Xi’an 710054, China

**Keywords:** *Fructus arctii polysaccharides*, structural characterization, hypoglycemic activity

## Abstract

In recent years, the number of diabetes patients worldwide has been increasing daily, and more than 700 million people are in a prediabetic state. *Fructus arctii* exhibits notable anti-diabetic activity, but its active components remain unclear. In this study, a polysaccharide (FAP-W) was extracted and characterized using UV, FTIR, HPLC, NMR, AFM, the Congo red test, and SEM. FAP-W has a molecular weight of 1.99 × 10^4^ Da and mainly consists of α-d-glucopyranosyl-(1→2)-[β-d-fructofuranosyl-(1→2)]_10_-β-d-furanofructosyl units. Monosaccharide analysis revealed mannose, glucose, galactose, and arabinose in a 3.4:23.59:21.27:47.7 ratio. In insulin-resistant HepG2 cells, FAP-W significantly increased glucose consumption, enhanced glycogen content, and elevated HK and PK activities. It also decreased TG, MDA, and ROS levels while improving SOD activity. These results suggest that FAP-W ameliorates insulin resistance, regulates glucose–lipid metabolism, and alleviates oxidative stress, indicating its potential as a functional food or therapeutic candidate for diabetes.

## 1. Introduction

Diabetes mellitus (DM) is a chronic metabolic disorder characterized by persistent hyperglycemia due to impaired insulin secretion, insulin resistance, or both [1]. It is among the most prevalent non-communicable diseases worldwide, posing a significant global health and economic burden. According to the International Diabetes Federation, approximately 537 million adults had diabetes in 2021, accounting for 10.5% of the global population, and this number is projected to increase to 783 million by 2045, resulting in an estimated 6.7 million diabetes-related deaths annually [2,3]. Diabetes is associated with severe complications, including cardiovascular disease, retinopathy, nephropathy, kidney failure, neurological disorders, and diabetic foot [4,5].

In addition to the rising number of diagnosed patients, a substantial proportion of the global population remains in a prediabetic state, characterized by impaired glucose tolerance (IGT) or impaired fasting glucose (IFG), but not yet meeting the threshold for pharmacological intervention. For instance, Rooney et al. [6] estimated that in 2021, approximately 464 million adults worldwide had IGT and 298 million had IFG. Since most of these individuals are not suitable for or do not require drug treatment, dietary management and functional foods have become promising strategies to delay or prevent progression to overt diabetes [7].

In this context, medicinal and edible plants, traditionally referred to as “homology of medicine and food” resources, have gained increasing attention. Such natural materials are valued for their high safety, accessibility, and abundance of bioactive components, which can beneficially regulate glucose and lipid metabolism, alleviate oxidative stress, and maintain metabolic homeostasis. Among them, plant-derived polysaccharides have been widely studied due to their potent hypoglycemic, antioxidant, and anti-inflammatory activities. A variety of natural products such as *Reynoutria japonica* Houtt. polysaccharides, *Astragalus membranaceus* (Fisch.) Bunge polysaccharides, *Poria cocos* (Schw.) Wolf polysaccharides, *Siraitia grosvenorii* polysaccharide, and other natural products have the activities of improving insulin resistance (IR), regulating the activity of glycolipid metabolizing enzymes, and lowering blood lipids and fasting blood glucose [8,9,10,11].

*Fructus arctii*, the dried mature fruit of *Arctium lappa* L. (Asteraceae), is a representative medicinal and edible plant widely distributed in East Asia [12,13,14]. Pharmacological studies have demonstrated its multiple biological activities, including antioxidant, anti-inflammatory, and hypoglycemic effects [15,16,17,18]. Polysaccharides are considered one of its major active components. Recent studies suggest that *Fructus arctii* polysaccharides may regulate lipid metabolism, modulate gut microbiota, and alleviate oxidative stress [19,20,21]. However, to date, studies on the structure and hypoglycemic effects of *Fructus arctii* polysaccharides have been relatively limited, with most research focusing on other biological activities [22]. Investigating their structure and bioactivities is therefore critical for understanding their potential in glucose metabolism regulation and for developing them as functional food ingredients or natural therapeutic agents for diabetes management.

In this study, we aimed to extract and characterize *Fructus arctii* polysaccharides, with a focus on their monosaccharide composition, chain structure, and glycosidic linkages. The effects on insulin-resistant human hepatocellular carcinoma (IR-HepG2) cells were evaluated by assessing glucose consumption, glycogen content, key glycolytic enzyme activities, and oxidative stress markers. This study not only addresses the current gaps in the structural elucidation of FAP-W and research on its hypoglycemic activity, but also provides a scientific basis for developing *Fructus arctii* polysaccharides into functional hypoglycemic foods or therapeutic agents.

## 2. Results

### 2.1. Structural and Physicochemical Features of FAP-W

*Fructus arctii* polysaccharide–water extract (FAP-W) exhibited a total sugar content of 89.1%, alongside a minor protein content of 1.58%. The glucuronic acid and reducing sugar contents were measured at 2.1% and 7.57%, respectively. Additionally, FAP-W demonstrated a particle size of 10.63 nm and a zeta potential of 98 mV. The ultraviolet (UV) full-wavelength scanning results (Figure S1) showed no obvious absorption peaks at 260 or 280 nm, indicating that FAP-W contained negligible amounts of nucleic acids and proteins, suggesting high purity of the polysaccharide preparation.

### 2.2. Structural Characterization of FAP-W

#### 2.2.1. Atomic Force Microscope (AFM)

**Figure 1:**
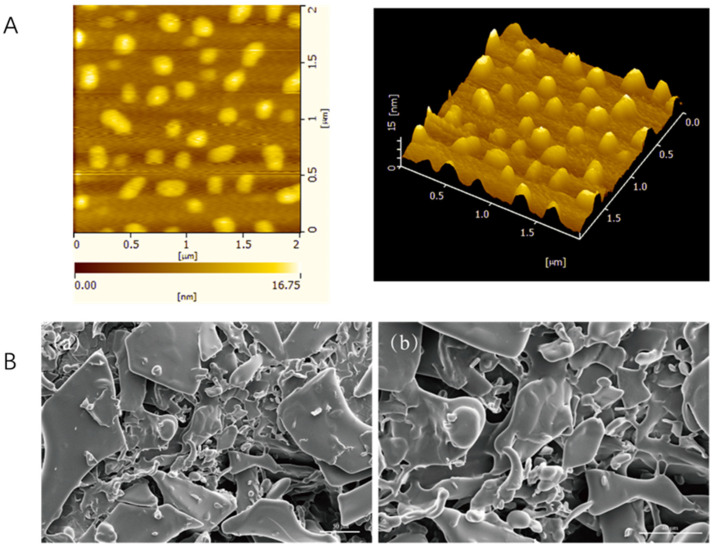
Morphological characterization of FAP-W. (**A**) Atomic force microscopy analysis; (**B**) SEM images at 1000× magnification (**a**) and 2000× magnification (**b**).

The atomic force microscopy scanning results of FAP-W are shown in Figure 1A. It can be observed that the surface of FAP-W is relatively smooth, exhibiting uniformly distributed peak-like aggregates with a height of approximately 16.75 nm. These results suggest that the FAP-W molecules may be composed of multiple polysaccharide chains tightly packed together, forming a dense and ordered aggregate structure.

#### 2.2.2. Scanning Electron Microscope (SEM)

The SEM results of FAP-W are presented in Figure 1B. As can be seen in the SEM image, the polysaccharides were aggregated in fragments with a smooth surface and a loose texture.

#### 2.2.3. Molecular Weight

The molecular weight of FAP-W was calculated by fitting the glucan standard curve. Based on the experimental results, the number-average molecular weight (Mn), weight-average molecular weight (Mw), and peak molecular weight (Mp) of FAP-W were determined to be 1.42 × 10^4^ Da, 1.99 × 10^4^ Da, and 1.69 × 10^4^ Da, respectively.

#### 2.2.4. Monosaccharide Composition Analysis

**Figure 2:**
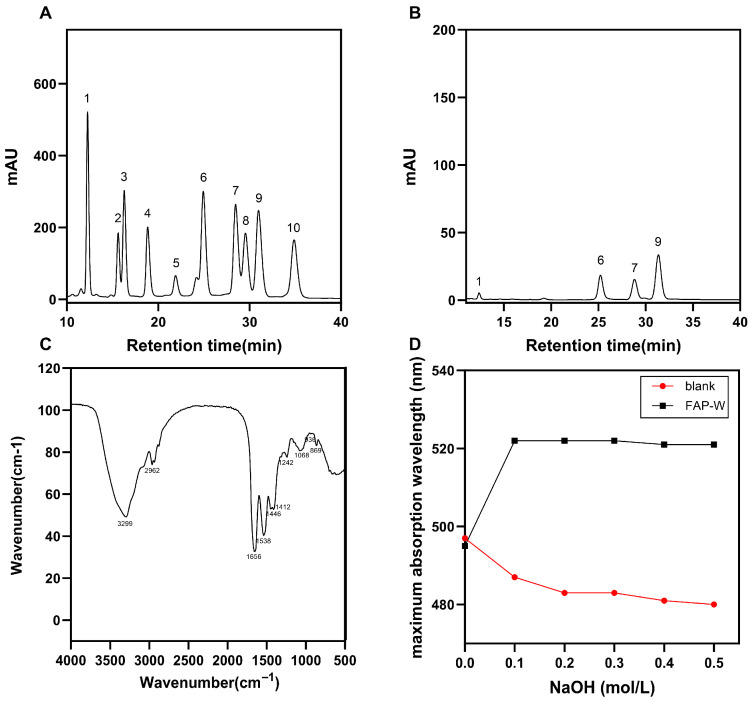
Characterization of FAP-W: physicochemical, monosaccharide, and spectral analyses. (**A**) FT-IR spectrum; (**B**) Congo Red Test; (**C**) HPLC chromatogram of monosaccharide standard, 1. mannose; 2. ribose; 3. rhamnose; 4. glucuronic acid; 5. galacturonic acid; 6. glucose; 7. galactose; 8. xylose; 9. arabinose; 10. fucose; (**D**) HPLC chromatogram, 1. mannose; 6. glucose; 7. galactose; 9. arabinose.

The liquid chromatograms of the standard monosaccharide and FAP-W are depicted in Figure 2A,B. By comparing with various standards and calculating the molar ratios of different monosaccharides in FAP-W, it can be determined that FAP-W primarily comprises mannose, glucose, galactose, and arabinose. The most abundant monosaccharides are arabinose and glucose, followed by galactose, while mannose is the least abundant with a molar ratio of 3.4:23.59:21.27:47.7.

#### 2.2.5. Fourier Infrared Spectroscopy (FT-IR) Spectroscopy Analysis

The results of FT-IR of FAP-W are shown in Figure 2C The observed peaks in the range of 3600 to 3200 cm^−1^ and 1668 to 1631 cm^−1^ suggest that the compound is a carbohydrate. The prominent peak at 3299 cm^−1^ corresponds to the stretching vibration of the O-H bond, while the smaller peak at 2962 cm^−1^ can be attributed to the stretching vibration of methyl and methylene C-H bonds. The peak at 1656 cm^−1^ arises from the stretching vibration of -CO, while the absorption peak at 1446 cm^−1^ is attributed to the bending deformation vibration of the C-H bond. The presence of a pyranose ring is indicated by the observed peaks at 1068 cm^−1^, 1051 cm^−1^, and 1026 cm^−1^. The absorption peak at 869 cm^−1^ indicates the presence of a β-configuration glycosidic bond and the absorption peak at 936 cm^−1^ corresponds to an α-d-glucopyranosyl group [23,24,25].

#### 2.2.6. Congo Red Test Analysis

The Congo red test results of FAP-W are shown in Figure 2D. The maximum absorption wavelength (λ_max_) of the FAP-W solution initially increased and then stabilized as the concentration of NaOH solution gradually increased, without any significant decrease. The results of the Congo red test indicate that there is no triple helix structure in FAP-W [26,27,28].

#### 2.2.7. Nuclear Magnetic Resonance (NMR) Spectroscopy Analysis

In the Proton Nuclear Magnetic Resonance (^1^H-NMR) spectra, the chemical shifts (δ, ppm) of burdock neutral polysaccharides ranged from 3.40 to 5.50 ppm (Figure 2A), showing typical characteristics of polysaccharides. There is a signal at δ 5.37 ppm, which is a glycosidic allosteric proton in the α configuration. The rest of the peaks were between δ_H_ 4.16–4.26 ppm, δ_H_ 4.02–4.09 ppm, and δ_H_ 3.62–3.97 ppm, where δ_H_ 4.16–4.26 ppm was the H-3 value for fructose. In the Carbon-13 Nuclear Magnetic Resonance (^13^C-NMR) spectrum (Figure 2B), stronger signals of 103.77, 81.17, 76.95, 69.78, 62.26, and 60.62 can be found, which are attributed to fructose residue [29,30].

In Heteronuclear Single Quantum Coherence (HSQC) spectroscopy (Figure 2C), δ_C_ 60.56/δ_H_ 3.72 for the signal of fructose residue C-1. While δ_C_ 77.19/δ_H_ 4.22, δ_C_ 74.44/δ_H_ 4.16, δ_C_ 81.06/δ_H_ 3.85 and δ_C_ 62.18/δ_H_ 3.75 signaled fructose residues C-3, C-4, C-5 and C-6, respectively. In addition, the secondary signal at δ_C_ 92.19/δ_H_ 5.4 was attributed to the glucose residue C-1 signal. And δ_C_ 71.54/δ_H_ 3.60, δ_C_ 72.34/δ_H_ 3.82 and δ_C_ 69.44/δ_H_ 3.41 were C-2, C-3, C-4 of glucose residues [31]. Further elucidation of the arrangement of monosaccharides in FAP-W by HMBC spectroscopy (Figure 2D). The allosteric proton H-1 (δ_H_ 5.4 ppm) of the glucosyl residue showed a strong cross peak with C-2 (δ_C_ 103.56 ppm) of the fructosyl residue, suggesting the existence of a 1,2 linkage between glucose and fructose. The strong correlation between fructose residue C-2 (δ_C_ 103.13 ppm) and fructose residue H-1 (δ_H_ 3.7 ppm, δ_H_ 3.87 ppm) demonstrated the existence of 2,1 linkages between fructose residues [32].

**Figure 3:**
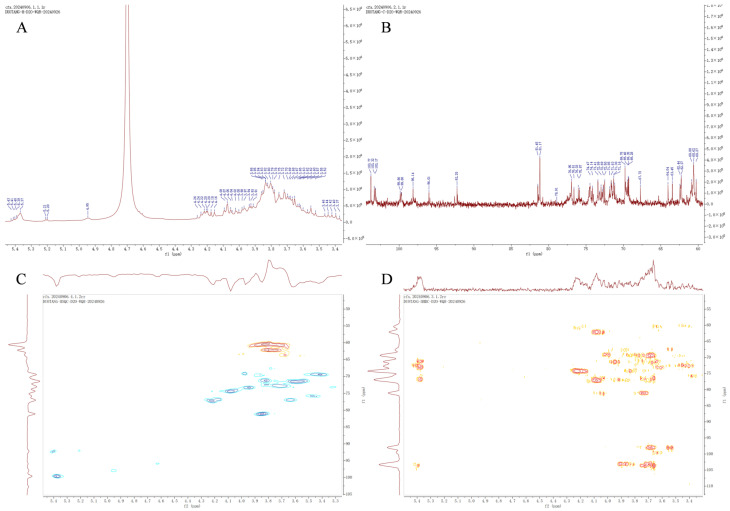
NMR spectral analysis of FAP-W. (**A**) ^1^H spectrum; (**B**) ^13^C spectrum; (**C**) HSQC spectrum. (**D**) HMBC spectrum.

Based on the above findings, FAP-W is primarily composed of α-d-glucopyranosyl-(1→2)-[β-d-fructofuranosyl-(1→2)]_10_-β-d-furanofructosyl units (Figure 3).

### 2.3. Hypoglycemic Activity of FAP-W in IR-HepG2 Cells

#### 2.3.1. Validation of the Insulin Resistance Model

**Figure 4:**
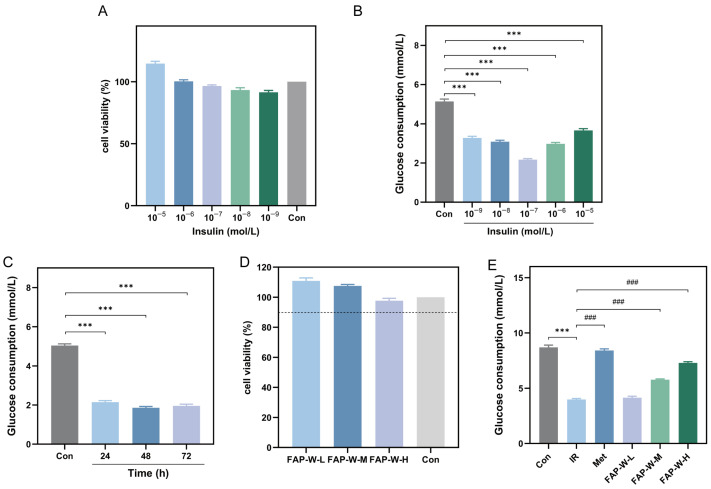
Effects of FAP-W on cell viability and glucose consumption in insulin-induced HepG2 cell model. (**A**) The effect of various concentrations of insulin on cell viability; (**B**) changes in glucose consumption of HepG2 cells under the influence of different insulin concentrations (compared with Control group, *** *p* < 0.001); (**C**) changes in glucose consumption in HepG2 cells under the influence of different action time (compared with Control group, *** *p* < 0.001); (**D**) the effect of different doses of FAP-W on cell viability (In the figure, the dashed line indicates a survival rate of 90%.); (**E**) the effect of FAP-W on glucose consumption of insulin-induced HepG2 cell model (Compared with Control group, *** *p* < 0.001; compared with IR group, ### *p* < 0.001).

The HepG2 cells were treated with various concentrations of insulin for 48 h in order to determine the optimal insulin concentration for establishing the IR-HepG2 model. The lower figure displays the cell viability results of HepG2 cells treated with varying concentrations of insulin. According to Figure 4A, it is evident that each concentration exhibits a cell survival rate exceeding 90%, indicating the absence of cytotoxicity at all tested insulin concentrations. Consequently, these specific insulin concentrations can be used for constructing an IR-HepG2 cell model.

The glucose consumption of HepG2 cells was assessed by exposing the cells to insulin at different concentrations and durations. As shown in Figure 4B, compared to the control group, the glucose consumption of HepG2 cells treated with different concentrations of insulin was significantly reduced. Among them, the glucose consumption of HepG2 cells treated with 1 × 10^−7^ mol/L insulin reached the lowest level, only 42.22% of that of the control group.

After determining the initial concentration for optimal insulin modeling, the optimal modeling time was further screened. Glucose consumption was assessed after treating HepG2 cells with 1 × 10^−7^ mol/L insulin for 24 h, 48 h, and 72 h, and the corresponding results are depicted in Figure 4C. The glucose consumption in HepG2 cells was significantly reduced after different durations of insulin treatment compared to the Control group. At 48 h, the glucose consumption of HepG2 cells reached its lowest point, accounting for only 36.91% of that in the Control group.

In conclusion, the optimal conditions for constructing HepG2 cell models with IR were identified at a concentration of 1 × 10^−7^ mol/L for 48 h.

#### 2.3.2. Effect of FAP-W on Cell Survival Rate and Glucose Consumption

The optimal dose of FAP-W was determined by subjecting HepG2 cells to various concentrations for 24 h. The results, depicted in Figure 4D, demonstrate that the cell survival rate of HepG2 cells remained above 90% across different drug concentrations, indicating a lack of significant inhibitory effects at low, medium, and high doses of FAP-W.

The glucose consumption of cells in each group is shown in Figure 4E. In the IR group, glucose consumption was significantly reduced. Treatment with MET increased glucose consumption by 111.31%, while medium- and high-dose FAP-W increased it by 45.23% and 83.42%, respectively. Compared to MET, medium-dose FAP-W achieved approximately 41% of its effect, while high-dose FAP-W reached about 75%. These results suggest that FAP-W effectively enhances glucose consumption in the IR-HepG2 cell model in a dose-dependent manner.

#### 2.3.3. Glycogen and Triglyceride Content (TG) Content

**Figure 5:**
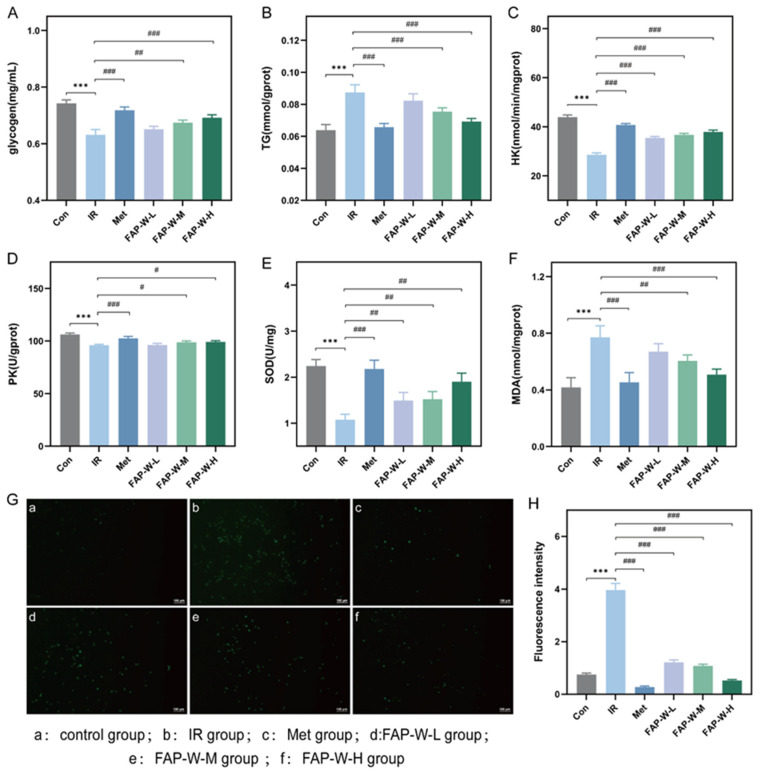
Effects of FAP-W on metabolic and oxidative stress markers in insulin-resistant HepG2 cells model. (**A**–**F**) The effect of FAP-W on the glycogen, TG, HK, PK, SOD and MDA content of IR-HepG2 cell model (Compared with Control group, *** *p* < 0.001; compared with IR group, ### *p* < 0.001, ## *p* < 0.01 # *p* < 0.05,); (**G**) Content of ROS (The figure shows a scale bar of 100 μm.); (**H**) Quantitative analysis of ROS fluorescence.

The glycogen content in each group of cells is shown in Figure 5A. The glycogen content in HepG2 cells of the IR group was significantly lower than that of the control group, decreasing by 14.86%. Compared to the IR group, the glycogen content in the MET group, as well as in the medium- and high-dose FAP-W groups, was significantly increased, with MET increasing glycogen by 14.29%, and the medium- and high-dose FAP-W increasing it by 6.35% and 9.52%, respectively. Compared to MET, medium-dose FAP-W achieved approximately 44% of MET’s effect, while high-dose FAP-W reached about 67%. These results indicate that these treatments can effectively enhance glycogen storage in the IR-HepG2 cell model.

The triglyceride content of cells in each group is shown in Figure 5B. The TG levels in HepG2 cells of the IR group were significantly higher than those of the control group, increasing by 37.50%. However, the MET group, as well as the medium- and high-dose FAP-W groups, showed a significant reduction in TG levels, with MET decreasing TG by 25.29%, and the medium- and high-dose FAP-W decreasing it by 14.77% and 22.73%, respectively. Compared to MET, medium-dose FAP-W achieved approximately 58% of MET’s effect, while high-dose FAP-W reached about 90%. These results suggest that these treatments can improve lipid metabolism under IR conditions.

#### 2.3.4. Hexokinase (HK) and Pyruvate Kinase (PK) Activity

The HK activity of cells in each group is shown in Figure 5C. Compared to the control group, the HK content in HepG2 cells of the IR group was significantly decreased by 34.90%. Both the MET group and the medium- and high-dose FAP-W groups showed significant increases in HK activity, with MET increasing it by 42.57%, and the medium- and high-dose FAP-W increasing it by 28.12% and 32.53%, respectively. Compared to MET, medium-dose FAP-W achieved approximately 66% of MET’s effect, while high-dose FAP-W reached about 76%. These results indicate that these treatments can improve glycolytic capacity in the IR-HepG2 cell model.

The PK activity of cells in each group is shown in Figure 5D. The PK content in HepG2 cells of the IR group was significantly lower than that of the control group, decreasing by 9.80%. Both the MET group and the medium- and high-dose FAP-W groups showed significant increases in PK activity, with MET increasing it by 6.94%, and the medium- and high-dose FAP-W increasing it by 3.04% and 3.47%, respectively. Compared to MET, medium-dose FAP-W achieved approximately 44% of MET’s effect, while high-dose FAP-W reached about 50%. These results suggest that these treatments can effectively enhance glycolytic flux in the IR-HepG2 cell model.

#### 2.3.5. Effect of FAP-W on Oxidative Damage in the IR-HepG2 Model

Superoxide dismutase (SOD) plays a crucial role in eliminating excessive reactive oxygen species and protecting cells from oxidative damage. The content of SOD in each group of cells is shown in Figure 5E. In the IR group, the SOD levels in HepG2 cells were significantly lower than those in the control group, decreasing by 51.79%. Treatment with MET and all doses of FAP-W significantly increased SOD content, with MET increasing it by 101.85%, and medium- and high-dose FAP-W increasing it by 40.74% and 75.93%, respectively. Compared to MET, medium-dose FAP-W achieved approximately 40% of MET’s effect, while high-dose FAP-W reached about 75%.

Malondialdehyde (MDA), the ultimate product of lipid peroxidation, is an important indicator to reflect the oxidative stress of the body. The content of MDA in each group of cells is shown in Figure 5F. The MDA levels in HepG2 cells of the IR group were significantly higher than those of the control group, increasing by 85.71%. Compared to the IR group, the MDA content in the MET group and the medium- and high-dose FAP-W groups was significantly reduced, with MET decreasing MDA by 41.56%, and the medium- and high-dose FAP-W decreasing it by 22.07% and 33.77%, respectively. Compared to MET, medium-dose FAP-W achieved approximately 53% of MET’s effect, while high-dose FAP-W reached about 81%. These results suggest that these treatments can effectively alleviate lipid peroxidation in the IR-HepG2 cell model.

Hyperglycemia and IR play pivotal roles in the pathogenesis of atherosclerosis and its complications. Metabolic abnormalities can lead to excessive generation of reactive oxygen species (ROS), when reaching a certain threshold, trigger endothelial dysfunction and inflammation, thereby promoting diabetic vascular disease [33]. Therefore, by assessing intracellular ROS content, we can comprehensively understand cellular changes under conditions of IR and investigate mechanisms associated with drug efficacy.

The results of cellular ROS detection are shown in Figure 5G, with the corresponding quantitative analysis of ROS fluorescence presented in Figure 5H. The control group (a) exhibited weak green fluorescence, indicating a low level of ROS in HepG2 cells under normal conditions. In contrast, the IR group (b) displayed strong and abundant green fluorescence, reflecting an increase in ROS content induced by insulin in HepG2 cells. The green fluorescence intensity in the MET group (c) and the medium- and high-dose FAP-W groups (e,f) was lower than in the IR group, and the fluorescence intensity in the medium- and high-dose FAP-W groups was slightly higher than in the MET group. These findings indicate that these treatments can improve oxidative damage induced by IR in HepG2 cells.

In summary, FAP-W may alleviate IR not only by reducing oxidative stress but also by promoting glycolysis, thereby mitigating hyperglycemia caused by IR.

## 3. Discussion

In recent years, plant polysaccharides have gained widespread attention due to their high safety, structural diversity, and multiple bioactive functions. Modern research has demonstrated that plant polysaccharides can exert hypoglycemic effects via various mechanisms, and their biological activities are strongly influenced by structural characteristics such as molecular weight, monosaccharide composition, and glycosidic bond types. For instance, polysaccharides with higher molecular weights generally exhibit stronger anti-diabetic activity, highlighting the significance of studying both structure and pharmacological functions for their development and utilization [34,35,36].

In this study, a comprehensive set of analytical techniques was employed to investigate the structural features and hypoglycemic effects of *Fructus arctii* polysaccharide (FAP-W) and to assess its influence on the physiological and biochemical properties of insulin-resistant HepG2 (IR-HepG2) cells. UV full-wavelength scanning revealed no significant absorption peaks at 260 and 280 nm, indicating the absence of nucleic acids and proteins in FAP-W. IR and NMR spectroscopy analyses showed that FAP-W is mainly composed of α-d-glucopyranosyl-(1→2)-[β-d-fructofuranosyl-(1→2)]_10_-β-d-furanofructosyl. Dextran gel permeation chromatography determined its molecular weight to be 1.99 × 10^4^ Da. Monosaccharide composition analysis indicated that FAP-W primarily consists of mannose, glucose, galactose, and arabinose in a molar ratio of 3.4:23.59:21.27:47.72. Atomic force microscopy and Congo red assays suggested the absence of a triple helix structure, while scanning electron microscopy revealed aggregated fragments with smooth surfaces and loose textures.

Molecular weight is a critical factor influencing polysaccharide bioactivity [35]. According to previous studies, polysaccharides can be classified into three categories based on their relative molecular weight: high (>100 kDa), medium (10–100 kDa), and low (<10 kDa) [37]. Polysaccharides of medium molecular weight generally exhibit higher bioavailability, effectively entering cells or binding to cell surface receptors, thereby modulating glucose metabolism more efficiently [38]. With a molecular weight of 1.99 × 10^4^ Da, FAP-W is likely to possess favorable bioavailability, facilitating effective receptor interactions and glucose regulation. Zeng et al. (2023) demonstrated that *Lycium barbarum* polysaccharides with medium (≈7.5 kDa) and high (≈46.2 kDa) molecular weights exhibited markedly stronger antioxidant and immunomodulatory activities than those with low molecular weight (≈1.9 kDa) [39].

Monosaccharide composition also plays a pivotal role in hypoglycemic activity. Many hypoglycemic polysaccharides contain arabinose, galactose, and glucose [40]. In diabetes and its prediabetic states, oxidative stress is recognized as one of the key pathological factors contributing to insulin resistance and pancreatic β-cell dysfunction. Excessive ROS can damage cellular membranes, proteins, and DNA, thereby disrupting insulin signaling pathways and leading to abnormal glucose regulation. Notably, arabinose contributes to antioxidant and immunomodulatory effects, which can alleviate inflammation by reducing oxidative stress and promoting immune balance [41]. FAP-W is enriched in arabinose, suggesting its potential for both hypoglycemic activity and mitigation of oxidative stress. For example, L-arabinose–rich sugar beet pulp demonstrates notable potential in managing diabetes, regulating blood glucose levels, and exerting antioxidant activity [42]. FAP-W is mainly composed of α-d-glucopyranosyl-(1→2)-[β-d-fructofuranosyl-(1→2)]_10_-β-d-furanofructosyl. The specific combination of an α-glucose backbone with β-fructose side chains may form a flexible spatial structure, promoting interactions with cell surface receptors or metabolic enzymes, activating key enzymes, and enhancing glucose utilization [35]. This structural arrangement may also improve antioxidant capacity, reduce ROS accumulation, and indirectly enhance insulin sensitivity and lipid metabolism [36].

HK and PK are central glycolytic enzymes that catalyze the phosphorylation of glucose to glucose-6-phosphate (G6P) and the conversion of phosphoenolpyruvate (PEP) to pyruvate, respectively. By governing these key rate-limiting steps, HK and PK are critical for the regulation of blood glucose homeostasis, cellular energy metabolism, and insulin sensitivity. Dysregulation of their activities has been closely associated with hyperglycemia and insulin resistance, hallmark features of type 2 diabetes. Therefore, modulation of HK and PK function represents a promising strategy for the development of hypoglycemic interventions and functional bioactive compounds aimed at improving glucose metabolism [43,44]. FAP-W significantly increased glucose consumption and glycogen synthesis and enhanced the activities of key glycolytic enzymes, HK and PK. It also effectively reduced TG accumulation and alleviated oxidative stress, as evidenced by decreased ROS and MDA levels, alongside elevated SOD activity. These findings indicate that FAP-W not only exerts direct hypoglycemic effects but also mitigates oxidative stress, consistent with previous reports linking oxidative stress to insulin resistance and lipid metabolism dysregulation [45]. The cellular effects observed align closely with FAP-W’s structural features, further validating its bioactivity. Furthermore, FAP-W was compared with the commonly used antidiabetic drug MET. The results demonstrated that FAP-W could partially mimic the pharmacological effects of MET in a dose-dependent manner. Although its efficacy was relatively lower than that of MET, FAP-W still exhibited notable antidiabetic activity. Moreover, as a natural polysaccharide derived from *Fructus arctii*, FAP-W exhibits high safety and low toxicity, suggesting its potential suitability for long-term consumption compared with MET.

FAP-W exhibited significant hypoglycemic activity in the IR-HepG2 cell model. High-dose FAP-W increased glucose consumption to approximately 75% of MET efficacy, while medium-dose achieved about 41%, demonstrating a clear dose-dependent effect. Its glucose consumption level was comparable to that of *Nelumbo nucifera lotus plumul* polysaccharide NNP-2 (approximately 80% of MET) [46]. Regarding key indicators of glucose metabolism, the high-dose FAP-W group achieved glycogen content, HK, and PK activities reaching 67%, 67%, and 50% of MET values, respectively, whereas the medium-dose group showed 44%, 66%, and 44%. In comparison, *Siraitia grosvenorii* polysaccharides exhibited improvement ranges of 43–64% for glycogen, 53–87% for HK, and 15–79% for PK, indicating that FAP-W exerts regulatory effects on glucose metabolism comparable to those of *Siraitia grosvenorii* polysaccharides [11].

Structurally, FAP-W has a low molecular weight of 1.99 × 10^4^ Da, with its monosaccharide composition primarily consisting of arabinose, galactose, and glucose. This polysaccharide contains mixed α/β glycosidic bonds and lacks a triple-helix structure, sharing similar structural characteristics with *Siraitia grosvenorii* polysaccharides and *Imperatae Rhizoma* polysaccharides known for their hypoglycemic activity [11,47]. Notably, the lower molecular weight and simpler glycan structure of FAP-W may facilitate faster cellular uptake and more efficient interaction with glucose metabolism-related targets. These structural features likely contribute to its superior capacity to enhance glucose consumption and glycogen synthesis compared with higher molecular weight or more complex polysaccharides [35].

FAP-W, a polysaccharide derived from *Arctium lappa*, has received limited attention regarding its antidiabetic potential. Most previous studies have focused on its phenolic compounds or arctiin, while the hypoglycemic activity of its polysaccharide fraction remains largely unexplored.

In this work, the structural characteristics of FAP-W were systematically elucidated. The polysaccharide exhibited a backbone of α-d-glucopyranosyl-(1→2)-[β-d-fructofuranosyl-(1→2)]_10_-β-d-furanofructosyl., a molecular weight of 1.99 × 10^4^ Da, and an arabinose-rich composition. These findings provide valuable insights into the structure–activity relationship of burdock polysaccharides. Unlike studies limited to α-glucosidase inhibition assays, this study preliminarily investigated the hypoglycemic activity of FAP-W. The results showed that FAP-W enhanced glycolytic enzyme activities (HK and PK), alleviated oxidative stress by decreasing ROS and MDA levels while increasing SOD activity, and reduced intracellular lipid accumulation. A comparative analysis with metformin (MET) further demonstrated that high-dose FAP-W could partially mimic the hypoglycemic effect of MET, emphasizing its pharmacological relevance. Overall, FAP-W exhibits distinct structural features and multiple beneficial biological activities. These results suggest that FAP-W has potential as a safe and natural modulator of glucose metabolism, providing a new foundation for further research on *Arctii Fructus* polysaccharides.

**Figure 6:**
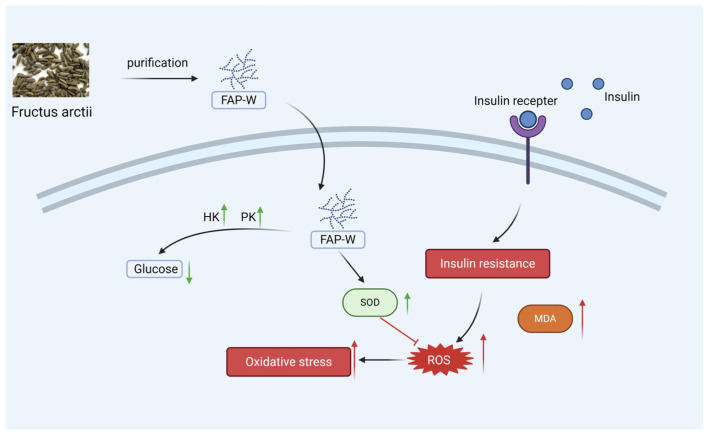
Mechanism of FAP-W in alleviating insulin resistance. Prolonged exposure to insulin results in reduced sensitivity of insulin receptors and glucose accumulation, which consequently elevates the levels of reactive oxygen species (ROS) and triggers oxidative stress. Nevertheless, FAP-W enhances superoxide dismutase (SOD) activity, decreases ROS levels, and mitigates oxidative stress. Furthermore, FAP-W increases the activities of hexokinase (HK) and pyruvate kinase (PK), thereby promoting the glycolytic pathway and exhibiting hypoglycemic activity. In the figure, red arrows indicate damage, and green arrows indicate protection. Created with Biorender.

The molecular weight, monosaccharide composition, and glycosidic linkage structure of FAP-W may contribute to its hypoglycemic activity. Cell experiments demonstrated that FAP-W can effectively lower blood glucose and alleviate oxidative stress, and its potential mechanisms of action were predicted, as shown in Figure 6. FAP-W, as a functional food ingredient or natural therapeutic agent, demonstrates considerable potential in alleviating or mitigating insulin resistance and diabetes.

## 4. Materials and Methods

### 4.1. Materials and Chemicals

Potassium bromide, arabinose, rhamnose, xylose, mannose, glucose, fucose, galactose, galacturonic acid, uronic acid and ribose were procured from Shanghai Yuanye Biotechnology Co., Ltd. (Shanghai, China). Acetonitrile and chloroform were procured from ASC in their analytical grade of purity. The 1-phenyl-3-methyl-5-pyrazolone (PMP) was purchased from Shanghai Aladdin Biochemical Technology Co., Ltd. (Shanghai, China). Sodium hydroxide, hydrochloric acid, and Congo red were obtained from Sinopdrug Group Chemical Reagent Co., Ltd. (Shanghai, China). All other chemicals and reagents utilized were of analytical grade.

### 4.2. Extraction and Physicochemical Characterization of FAP-W

#### 4.2.1. Extraction of FAP-W

*Fructus arctii* was powdered and sieved, refluxed with petroleum ether and ethanol and dried. The *Fructus arctii* powder was subjected to ultrasound-assisted-water extraction and alcohol precipitation to obtain *Fructus arctii* polysaccharides aqueous solution. *Fructus arctii* polysaccharide was obtained by removing protein by sevag method and freeze-drying. After purification by DEAE-cellulose column chromatography and dextran column chromatography, refined *Fructus arctii* polysaccharides (FAP-W) was obtained by freeze-drying for subsequent experiments [18,48].

#### 4.2.2. Physicochemical Characterization of FAP-W

The total sugar content was determined using the phenol-sulfuric acid method [49], while the protein content was measured by the Coomassie Brilliant Blue assay [50]. The glucuronic acid content was analyzed according to the method described by Li et al. [51], and the reducing sugar content was determined following the procedure reported by Zhu et al. [52]. FAP-W was prepared into a 100 µg/mL solution, which was then used to determine particle size and zeta potential.

#### 4.2.3. High-Performance Gel Permeation Chromatography (HPLC)

The FAP-W and standard were dissolved in a solution with a concentration of 5 mg/mL, followed by centrifugation. The supernatant was filtered into the sample bottle. Subsequently, high performance gel permeation chromatography was conducted. The chromatographic conditions were as follows: The chromatographic system was a gel chromatography differential-multi-angle laser light scattering system. Depending on the nature of the polysaccharide, an exclusion column with a gel size corresponding to the molecular weight range is selected. The column temperature was set at 45 °C, the injection volume was 100 μL, the mobile phase was 0.1 M NaNO_3_, the isocratic elution was 0.4 mL/min, and the elution time was 100 min [53].

### 4.3. Structural Characterization

#### 4.3.1. Atomic Force Microscope

The FAP-W solution was prepared by adding 5 mg of FAP-W to 5 mL of ultrapure water, followed by stirring for 4 h and subsequent dilution to a concentration of 10 µg/mL. Then, a droplet of the solution (2 µL) was deposited onto freshly cut mica slices and allowed to air dry naturally. The determination was performed using contact mode imaging with a scan size of 2 μm × 2 μm and a scan rate of 1.2 Hz [54].

#### 4.3.2. Scanning Electron Microscope

The dried powder samples (FAP-W) were sputtered with gold under reduced pressure and determined by Scanning Electron Microscopy (FEI Verios 460, FEI, Hillsboro, OR, USA).

#### 4.3.3. Analysis of Monosaccharide Constitution

The appropriate amount of FAP-W was treated with 1 mL trifluoroacetic acid (4 mol/L), followed by hydrolysis in an oven at 120 °C for 2 h, and finally dried using nitrogen gas. The PMP-methanol solution (0.5 mol/L) and NaOH solution (0.3 mol/L) were added, followed by a 1-h water bath at 70 °C. Subsequently, the HCl solution (0.3 mol/L) was added, and three extractions were performed using chloroform. Finally, the aqueous layer was filtered and loaded onto the machine [55]. Chromatographic conditions: The chromatographic column was DionexCarbopacTMPA20 (Thermo Fisher Scientific, Sunnyvale, CA, USA) (3 × 150 mm) and the mobile phases were: A: H_2_O; B: NaOH (15 mm); C: NaOH (15 mm) and NaOAc (100 mm). The column temperature was maintained at 30 °C; the flow rate was set at 0.3 mL/min [56].

#### 4.3.4. UV–Vis Absorption Spectra

By dissolving 10 mg of FAP-W in 10 mL of distilled water, a polysaccharide solution with a final concentration of 1 mg/mL was obtained. The prepared FAP-W solution was subjected to ultraviolet scanning at wavelengths ranging from 200 nm to 400 nm, with detection performed at every 1 nm interval [11].

#### 4.3.5. FT-IR Spectroscopy

The functional groups of FAP-W were analyzed using Fourier infrared spectroscopy. An appropriate amount of dried FAP-W was mixed with KBr, thoroughly ground, pressed, and subsequently measured at 4000~400 cm^−1^ using a Fourier infrared spectrometer (Thermo Nicolet iS50, Thermo Fisher Scientific, Waltham, MA, USA) [55].

#### 4.3.6. Congo Red Test

The sodium hydroxide solutions with concentrations of 0.4, 0.8, 1.2, 1.6, and 2.0 mol/L were sequentially added to the wells of a 96-well plate. Subsequently, a polysaccharide solution with a concentration of 2 mg/mL and a Congo red solution with a concentration of 160 μg/mL were added and mixed for a duration of 10 min. The absorption spectra were measured within the range of 400 to 600 nm for each sample in order to determine the wavelength at which maximum absorption occurred under different NaOH concentrations.

#### 4.3.7. NMR Spectroscopy

50 mg of FAP-W powder was weighed and dissolved in 0.6 mL of D_2_O and thoroughly mixed by eddying. The resulting solution was then filtered through a microporous filter membrane with a pore size of 0.45 μm and transferred to an NMR tube for ^1^H NMR and ^13^C NMR via an NMR system (Bruker BioSpin, Billerica, MA, USA).

### 4.4. Assays of Hypoglycemic In Vitro

#### 4.4.1. Construction of an Insulin Resistance Model

The impact of insulin concentration on cell viability was investigated by Cell Counting Kit-8 (CCK-8) method. The cell density was adjusted to 5 × 10^4^ cells per well, and the cells were cultured overnight. Blank, control, and insulin groups were set. After the cells had adhered, the previous medium was discarded and replaced with serum-free medium for a duration of 10 h. Afterward, 10 μL of serum-free medium containing insulin at concentrations of 10^−9^, 10^−8^, 10^−7^, 10^−6^, and 10^−5^ mol/L were added to each experimental group. The 96-well plates were incubated in the incubator for the appropriate duration. Subsequently, 10 μL of CCK-8 solution was added to each well and allowed to incubate for 4 h. Finally, absorbance values were measured at a wavelength of 450 nm [57,58].

The HepG2 cells in the logarithmic growth phase were harvested and plated into 96-well plates at a concentration of 1 × 10^5^ cells/mL. The passage number of HepG2 cells is sixth. Blank and insulin groups were set. After 24 h of culture, the spent medium was discarded and replaced with complete medium for the blank control group. For the insulin group, serum-free medium containing insulin at concentrations of 1 × 10^−9^, 1 × 10^−8^, 1 × 10^−7^, 1 × 10^−6^ and 1 × 10^−5^ mol/L was added. Subsequently, the cells were incubated in a cell incubator for durations of 24 h, 48 h and 72 h, respectively. During this period, the fluid was changed every 24 h. After the incubation period, aspirate the supernatant and perform centrifugation, then collect the supernatant. Subsequently, the glucose content was determined following the instructions provided in the glucose measurement kit [58].

#### 4.4.2. Cytotoxicity Assay of FAP-W

The HepG2 cells in the logarithmic growth phase were harvested and seeded into 96-well plates at a concentration of 2 × 10^5^ cells/mL. Blank control group and drug group were set up. The drug treatment groups consisted of low, medium, and high doses of FAP-W with concentrations of 62.5, 250, and 1000 μg/mL, respectively. The impact of FAP-W at different doses on cell proliferation was evaluated using the CCK-8 assay (Beyotime Biotech Inc., Shanghai, China).

#### 4.4.3. Glucose Depletion Assay of FAP-W

The HepG2 cells in the logarithmic growth phase were harvested and plated into 6-well plates at a concentration of 2 × 10^5^ cells/mL. The experimental groups consisted of a blank control group, a model group, a MET group, and FAP-W low, medium, and high dose groups. A serum-free medium supplemented with 1 × 10^−7^ mol/L insulin was added to all wells, except for the blank group, followed by incubation for 48 h. After successful establishment of the model, serum-free medium was added to both the blank and model groups. The MET group received serum-free medium containing 1000 μg/mL metformin, while the low, medium, and high dose FAP-W groups were treated with serum-free medium containing 62.5, 250, and 1000 μg/mL FAP-W, respectively. The glucose content was determined using the glucose measurement kit method to assess the impact of FAP-W on glucose consumption in IR-HepG2 cells.

#### 4.4.4. Glucose Metabolism in IR-HepG2 Cells

The HepG2 cells were seeded in T25 flasks during the logarithmic growth phase, and the experimental groups were established according to the protocol outlined in Section 4.4.3. After incubating the cells for 24 h, the supernatant was discarded, the cells were washed with PBS, digested through trypsin, and then collected. After centrifugation at 1000 rpm for 3 min, the supernatant was discarded and the cellular precipitate was then washed with PBS once or twice, followed by discarding the supernatant through centrifugation again. The cell precipitate was resuspended in 1 mL of PBS and sonicated under ice water bath conditions. Subsequently, the final cell suspension supernatant was centrifuged at 4 °C and stored at −20 °C.

The protein content of the cell supernatant was quantified using the Bicinchoninic acid assay (BCA) assay, while the glycogen, HK and PK contents in the IR-HepG2 cells were determined utilizing the method specified in the glycogen, HK and PK assay kit (Nanjing Jiancheng Bioengineering Institute, Nanjing, China).

#### 4.4.5. Lipid Metabolism in IR-HepG2 Cells

The experimental procedure was performed as described in Section 4.4.4.

The TG contents in the IR-HepG2 cells were determined utilizing the method specified in the TG assay kit (Nanjing Jiancheng Bioengineering Institute, Nanjing, China).

#### 4.4.6. Oxidative Damage in IR-HepG2 Cells

The experimental procedure was performed as described in Section 4.4.4. The protein content of the cell supernatant was quantified using the BCA assay (Beyotime Biotech Inc., Shanghai, China), while the SOD and MDA content of IR-HepG2 cells were determined utilizing the method specified in the SOD and MDA assay kit (Nanjing Jiancheng Bioengineering Institute, Nanjing, China).

The HepG2 cells were seeded in T25 flasks during the logarithmic growth phase, and the experimental groups were established according to the protocol outlined in Section 4.4.3. After incubating the cells for 24 h, the supernatant was discarded, and 3 mL of 2′,7′-Dichlorofluorescin diacetate (DCFH-DA) (10 μmmol/L) was added. Following a 20-min incubation, the cells were washed three times with serum-free medium to effectively eliminate any residual DCFH-DA that did not penetrate into the cells. The sample was subsequently examined under a fluorescence inverted microscope, and the variation in ROS fluorescence intensity was observed upon excitation at wavelengths ranging from 450 to 490 nm.

### 4.5. Statistics Analysis

The graph was created using the Graphpad prism 8.0 software, and the experimental data were analyzed using SPSS statistical 26 software. To examine the significance level and distinction between the two groups, a one-way analysis of variance (ANOVA) test was utilized. The difference was deemed statistically significant when *p* < 0.05.

## 5. Conclusions

FAP-W was structurally characterized as a heteropolysaccharide composed mainly of Ara, Glc, Gal, and Man, with a molecular weight of 1.99 × 10^4^ Da. Structural analyses revealed a backbone of α-d-glucopyranosyl-(1→2)-[β-d-fructofuranosyl-(1→2)]_10_-β-d-fructofuranosyl residues without a triple-helix conformation, exhibiting a smooth and loose microstructure. Functionally, FAP-W significantly enhanced glucose consumption and glycogen synthesis in insulin-resistant HepG2 cells, increased the activities of key glycolytic enzymes (HK and PK), and reduced intracellular TG, ROS, and MDA levels, while elevating SOD activity. These findings indicate that FAP-W exerts both hypoglycemic and antioxidant effects, likely through the regulation of glucose metabolism and the alleviation of oxidative stress. In conclusion, FAP-W shows great potential for development as a functional food ingredient for the auxiliary management of prediabetes.

## Data Availability

Data are contained within the article.

## Supplementary Materials

The following supporting information can be downloaded at: https://www.mdpi.com/article/10.3390/molecules30224403/s1, Figure S1: UV full-spectrum analysis of FAP-W. Figure S2. Standard curves for compositional analysis of FAP-W.
