# Supplementary material for: Structural Characterization and In Vitro Hypoglycemic Activity of a Polysaccharides Obtained from Fructus arctii
Source: Molecules. 2025 Nov 14;30(22):4403. doi: 10.3390/molecules30224403 (PMC12655392; doi:10.3390/molecules30224403)
Supplement: Supplementary file 1 [file molecules-30-04403-s001.zip › molecules-3975806-supplementary.docx]

**
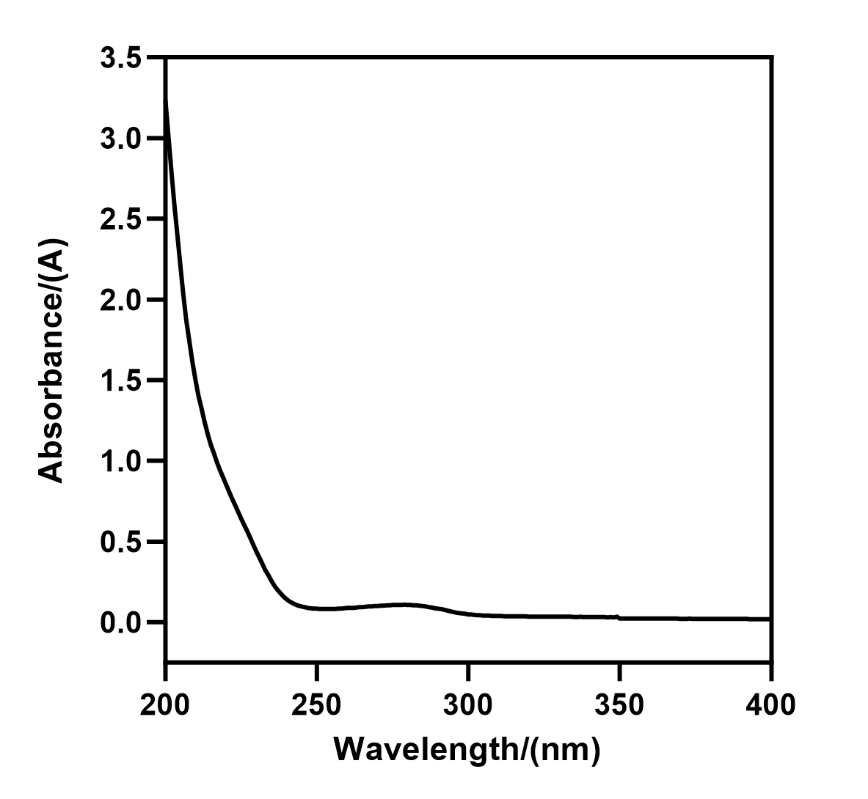
**

**Figure S1. UV full-spectrum analysis of FAP-W**


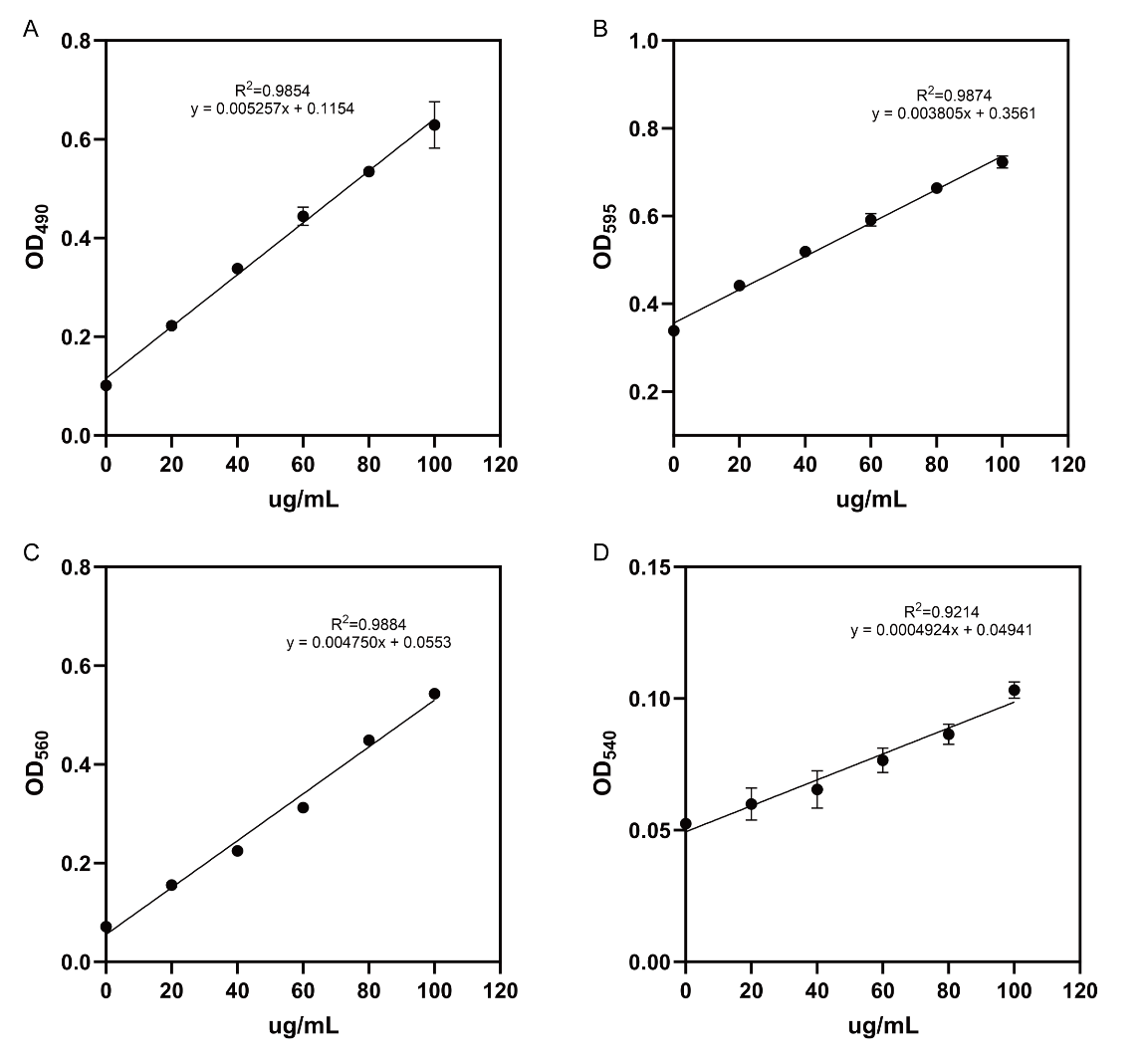


**Figure S2. Standard curves for compositional analysis of FAP-W.** (A) Total Sugar Standard Curve; (B) Protein content standard curve; (C) Glucuronic acid content standard curve; (D) Standard curve of reducing sugar content.

**(A)** **Total sugar content**

| FAP-W solution concentration | Y | X | Total sugar content/% |
| --- | --- | --- | --- |
| 20 μg/mL | 0.21025 | 17.89623 | 89.48113 |
| 60 μg/mL | 0.398583 | 53.43082 | 89.05136 |
| 100 μg/mL | 0.585833 | 88.76101 | 88.76101 |

**(B)** **Protein content**

| FAP-W solution concentration | Y | X | Protein content/% |
| --- | --- | --- | --- |
| 200 μg/mL | 0.372717 | 4.714912 | 2.357456 |
| 600 μg/mL | 0.385633 | 8.114035 | 1.352339 |
| 1000 μg/mL | 0.39345 | 10.17105 | 1.017105 |

**(C) Glucuronic acid content**

| FAP-W solution concentration | Y | X | Glucuronic acid content/% |
| --- | --- | --- | --- |
| 200 μg/mL | 0.084283 | 6.538194 | 3.269097 |
| 600 μg/mL | 0.09705 | 9.197917 | 1.532986 |
| 1000 μg/mL | 0.125083 | 15.03819 | 1.503819 |

**(D) Reducing sugar content**

| FAP-W solution concentration | Y | X | Reducing sugar content/% |
| --- | --- | --- | --- |
| 200 μg/mL | 0.0596 | 20.62686 | 10.31343 |
| 600 μg/mL | 0.0748 | 51.56377 | 8.593962 |
| 1000 μg/mL | 0.0681 | 37.95695 | 3.795695 |
